# Digital biomarker of mental fatigue

**DOI:** 10.1038/s41746-021-00415-6

**Published:** 2021-03-11

**Authors:** Vincent Wen-Sheng Tseng, Nachiappan Valliappan, Venky Ramachandran, Tanzeem Choudhury, Vidhya Navalpakkam

**Affiliations:** 1 Cornell University, Ithaca, NY USA; 2 Google Research, Mountain View, CA USA

**Keywords:** Predictive markers, Fatigue

## Abstract

Mental fatigue is an important aspect of alertness and wellbeing. Existing fatigue tests are subjective and/or time-consuming. Here, we show that smartphone-based gaze is significantly impaired with mental fatigue, and tracks the onset and progression of fatigue. A simple model predicts mental fatigue reliably using just a few minutes of gaze data. These results suggest that smartphone-based gaze could provide a scalable, digital biomarker of mental fatigue.

## Introduction

Mental fatigue is a key aspect of wellbeing^1–4^. Medical conditions^1,2,5^, sleep deprivation^6^ and prolonged task performance^7^ are some factors known to cause mental fatigue. It has been widely studied across medicine^1,2,5^, sleep research^6^, and mission-critical settings such as medical surgeries^8^ and aviation safety^9^. It is also important for digital wellbeing, where there is increasing societal concern over excessive time spent on screen and its potential negative impact on wellbeing^10,11^.

However, existing tests are subjective and/or time-consuming. Fatigue questionnaires such as the NASA Task Load Index (NASA-TLX)^12^ and Brief Fatigue Inventory (BFI)^13,14^ are subjective and susceptible to noise from self-reports. Gold-standard tests such as Psychomotor Vigilance Task (PVT)^15,16^ for alertness and N-back tests^17^ for working memory are time-consuming (e.g., standard PVT takes 10 min, though there exist less-validated shorter versions). Recent research also explored the use of facial features^18,19^ (such as eye blinks, head rotation, yawns) and text entry performance metrics^20^ for building smartphone-based driver and workplace fatigue detection systems. However, these studies did not use validated fatigue measures.

In this paper, using well-validated fatigue measures such as BFI and NASA-TLX, we leverage recent advances in accurate smartphone-based eye-tracking^21^ to test whether smartphone-based gaze can help detect mental fatigue. Given the pervasiveness of phones, a smartphone-based digital biomarker could provide a scalable and quick alternative for detecting mental fatigue.

**Fig. 1:**
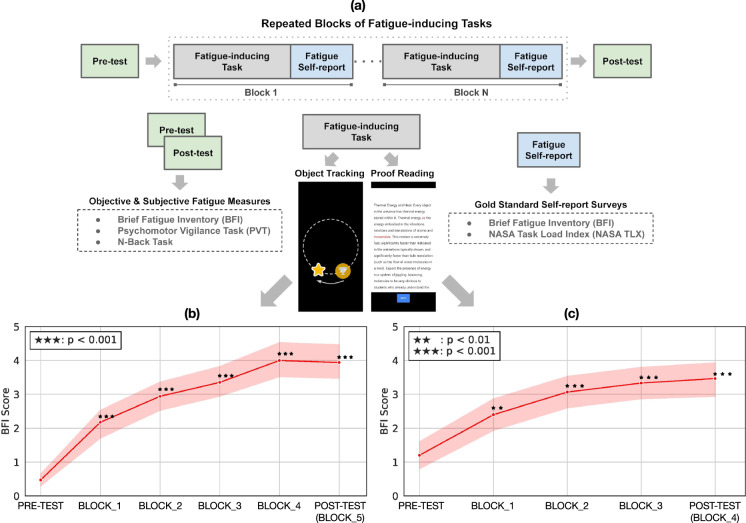
Study overview. **a** Experiment design. Participants performed a series of fatigue-inducing tasks over a prolonged duration. Gold-standard tasks were performed at the beginning (pre-test) and end of the study (post-test). Two types of fatigue-inducing tasks were used: an object-tracking task (study 1), and a proofreading task (study 2). **b**, **c** show the progression of fatigue scores across the 5 task blocks (in study 1) and 4 task blocks (in study 2), respectively. Error bands denote the Mean ± SEM (*n* = 17 and 15 participants for studies 1 and 2, respectively).

Participants performed a series of tasks over a prolonged duration of time (~1 h). As shown in Fig. 1a, at the beginning and end of the study, participants performed time-consuming gold-standard tests to measure the level of alertness, focused attention and mental fatigue. We call these pre- and post-test, respectively. Each block of tasks was followed by a short fatigue questionnaire (from the BFI, see Methods section) to measure the progression of mental fatigue during the study.

Two different types of fatigue-inducing tasks were used. Study 1 consisted of a language-independent, object-tracking task. 17 participants tracked an object that changed its shape randomly as it moved smoothly in a circular trajectory, and were asked to tap anywhere on the screen whenever they detected a particular shape. We call this the object-tracking task. Study 2 consisted of a language-dependent, reading task. 15 participants were asked to proofread English passages and detect/tap on words that had spelling or semantic errors. We call this the proofreading task.

As seen in Fig. 1b, c, repeated task performance leads to increased mental fatigue across both studies. Details on the fatigue scores, task and gaze features are shown in Table 1. The BFI score increases significantly from the beginning to the end of the study. NASA-TLX for mental demand also increases, though less sensitive than BFI, hence the remaining analysis in the paper focuses on BFI as the key fatigue measure. Consistent with previous work, we find that mental fatigue hurts task performance (drop in task accuracy, precision, recall and slower responses), though not all changes are significant. Analysis of gaze behavior shows significant gaze impairments with mental fatigue. Gaze features such as entropy, mean and standard deviation in gaze error (computed as the difference between gaze vs. actual target position) increase significantly with mental fatigue.

**Table 1:** Change in mental fatigue scores, task and gaze features for study 1.

Measures	No Fatigue	Fatigue	Statistical test	*p* value
	(Mean ± SEM)	(Mean ± SEM)		
BFI	0.47 ± 0.19	3.94 ± 0.54	t(16) = 6.39	<10^−5^
NASA-TLX	20.06 ± 5.74	28.41 ± 7.13	t(16) = 2.31	0.03
Task accuracy (%)	98.5 ± 0.3	97.4 ± 0.5	w(16) = 34	0.04
Task precision (%)	96.2 ± 0.7	93.8 ± 1.1	w(16) = 41	0.09
Task recall (%)	97.9 ± 0.6	96.1 ± 1.0	w(16) = 51	0.24
Task RT (ms)	484.66 ± 13.73	497.96 ± 0.65	w(16) = 50	0.21
Mean gaze error	0.38 ± 0.03	0.54 ± 0.05	w(16) = 10	0.002
Stddev gaze error	0.27 ± 0.02	0.40 ± 0.04	w(16) = 12	0.002
Gaze entropy	14.50 ± 0.14	15.20 ± 0.20	w(16) = 20	0.007

The first half of block 1 was considered as “No Fatigue”, and the last half of the final blocks (4,5) were considered as “Fatigue” condition. Statistical comparisons were performed with *n* = 17 participants using the two-tailed paired t-test or the Wilcoxon signed-rank test as denoted by t(.) and w(.) respectively.

**Fig. 2:**
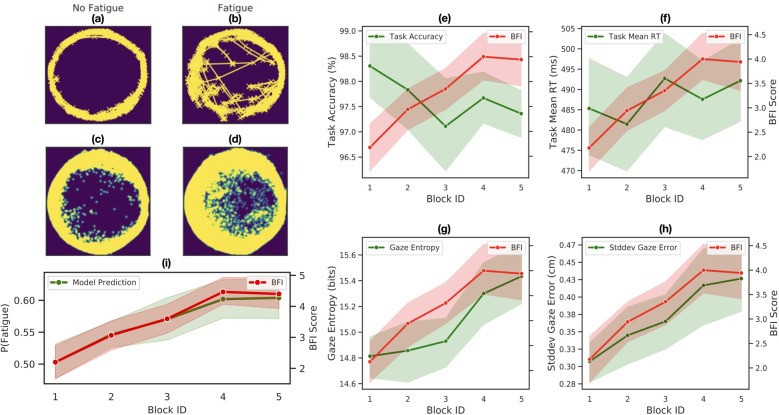
Progression of fatigue, gaze, and task performance features over time. **a**, **b** Sample gaze patterns from a single participant for the "No Fatigue'' vs. "Fatigue'' condition. **c**, **d** Corresponding population-level gaze heatmaps. **e**–**h**. Progression of task and gaze features over time. Error bands denote the Mean ± SEM (*n* = 17 participants). **i** Model prediction of the probability of fatigue over time, along with the self-reported BFI score.

Figure 2a, b shows an example gaze scanpath for one of the participants when they are fatigued vs. not. As seen here and the corresponding population-level gaze heatmaps (Fig. 2c, d), while participants’ gaze initially follows the circular trajectory of the object, under fatigue, gaze shows high errors or deviations from the circular trajectory. In addition to gaze differences between the fatigue vs. no-fatigue conditions, as shown in Fig. 2g, h, gaze features appear to track the onset and progression of fatigue during the course of the study.

To test whether fatigue can be predicted from objective task and gaze features, we built a simple binary classification model to classify each 75 seconds unit of a block (we call this “chunk”) for a given participant as fatigued or not. The Methods section describes the individual task and gaze features used in the model and the leave-one-out evaluation methodology. Gaze was found to be a strong predictor of mental fatigue—using just 75 seconds worth of gaze data on a new participant, the model achieved 80% accuracy (AUC 0.818) in detecting fatigue vs. no-fatigue, after collecting baseline normalization data. The model performance improved further upon using 150 sec of gaze data (AUC 0.839).

Gaze was found to be a better predictor of fatigue than (non-gaze) task performance based features (AUC of 0.818 ± 0.062 vs. 0.676 ± 0.078; *t*(14) = −1.586, *p* = 0.135; two-tailed paired *t*-test). Interestingly, the above gaze-based classification model is able to predict the probability of the user experiencing fatigue over the course of the study (Fig. 2i). Similar results were obtained for the proofreading task (study 2). A predictive model (similar to the model in study 1) shows that gaze can predict fatigue significantly better than task-based features (AUC of 0.833 ± 0.040 for gaze vs. 0.630 ± 0.038 for task; *t*(14) = −3.891, *p* = 0.002; two-tailed paired *t*-test).

We demonstrate that smartphone-based gaze is a strong predictor of mental fatigue; as well as tracks the onset and progression of mental fatigue. We validate these findings in two different experiments—using a language-independent object-tracking task, and a language-dependent proofreading task. These findings suggest that smartphone gaze could be a digital biomarker of mental fatigue with the potential for orders-of-magnitude scaling.

All the data in this study was collected in lab settings for research purposes with the participants’ explicit and fully informed consent. In addition, participants were allowed to opt out of the study at any point and request their data to be deleted, without affecting their compensation for participating in the study. This study has some limitations. While it is a proof-of-concept to demonstrate the potential of smartphone-gaze based fatigue detection, additional work including field trials and larger studies (across more diverse demographics) are required to explore other sources of fatigue such as sleep deprivation, stress, lack of motivation, longer duration of repeated tasks (over several hours), and extensive screen time.

Prior eye-tracking research^22,23^ used specialized and expensive hardware to show that sleep deprivation can lead to significant gaze impairments. Our study shows that fatigue-induced gaze impairments can be measured using just the smartphone’s selfie camera and without any additional hardware. This suggests the potential to scale sleep studies. Given the growing societal concern over large amount of time spent on screen (over 11 h/day^24^), smartphone-based measurements of mental fatigue could also offer smarter and timely interventions to improve digital wellbeing. Thus, smartphone-based digital biomarker of mental fatigue could unlock applications across improved sleep and wellness.

## Methods

### BFI

BFI questions were prompted during the pre-test, post-test, and after each block of fatigue-inducing tasks. Participants had to indicate the level of their fatigue by selecting a score on a 11-point Likert scale. Some questions in BFI ask about the level of an individual’s fatigue during the past 24 h. To avoid repetition, participants were prompted to answer these questions only once during the pre-test. For the remaining BFIs, participants were only required to answer the question regarding their fatigue level at the moment.

### NASA-TLX

The NASA-TLX questionnaire was prompted during the pre-test, post-test, and after each block of fatigue-inducing tasks. Questions for six different sub-scales, including Mental Demand, Physical Demand, Temporal Demand, Overall Performance, Effort, and Frustration Level, were presented in order. Participants were instructed to move the slider to indicate their response to each question on a range of 0 to 100.

### Eye-tracking

We used the smartphone-based eye-tracking model described in recent research^21^. The model was calibrated by asking participants to fixate on a green circular stimulus that appeared on a black screen. The stimulus appeared at random locations on the screen (dot calibration), or in a zigzag pattern from the upper left to lower right corner of the screen (zigzag smooth pursuit) for 30–60 s. Eye-tracking accuracy was computed as the Euclidean error between the true stimulus location and the estimated gaze locations on the phone screen, using a separate test set. The average model error (Mean ± SEM) across all participants is 0.420 ± 0.088 cm (with range [0.13, 0.74] cm) and 0.491 ± 0.065 cm (with range [0.17, 1.0] cm), for study 1 and 2 respectively. At a viewing distance of 25–40 cm, this translates to 0.6–1.1^∘^ angular accuracy.

### Participants

Participants aged 18 and above were recruited from a pool of user study volunteers who signed up through the Google User Experience Research portal^25^. Approximately 60% of participants identified themselves as male, all others as female across both the studies. Prior to the recruitment of participants, this study was reviewed by the Advarra Institutional Review Board (IRB) (Columbia, MD) and determined to be exempt from IRB oversight. Each participant provided their explicit and informed consent to data collection by reading and signing a study-specific participant agreement that informed them about collecting the front-facing camera feed for research analyses purposes, and the potential risks involved in performing gaze tasks for several minutes (e.g., eye strain, fatigue). Participants received monetary compensation for their time even if they did not complete the tasks, and retained the option to have their data deleted at any time. Studies were designed to be <1 h long and were conducted in lab settings (in groups of 5–6 people).

### Android app

Data were collected with a custom Android app. The app served two main purposes: (1) to display the stimulus along with task instructions on screen; (2) capture and store the front-facing camera feed, as well as user touch interactions on the screen.

### Fatigue model

We used a simple soft-margin kernel support vector machine (SVM) binary classifier to build the fatigue estimation model. The task and/or gaze features were normalized per participant and used as input features. Specifically, we found that best results were obtained by normalizing/scaling each feature *x* as (*x* − *μ*)/*μ* where *μ* is the mean value of the feature across blocks per participant. The model outputs a probability estimate that the user is in a fatigued state. We then used the same model to predict and track the onset of fatigue for a new user. The fatigue model performance (reported as the average AUC) was evaluated in a leave-one-out setting. In this setting, the model gets repeatedly evaluated for each participant by leaving their data out of the training set and the reported metrics are the average across all participants.

### Fatigue labels

For descriptive analyses, the fatigue labels were fixed across participants for both studies. Since the user’s fatigue level may change within a block, the first half of the first block was labeled as "No Fatigue”, and the last half of the final two blocks as "Fatigue”. For the fatigue model in Fig. 2, we refined/personalized the fatigue labels based on BFI scores for each participant. In particular, the blocks with lowest self-reported BFI scores were labeled as “No Fatigue” (similarly, highest BFI scores were labeled as “Fatigue”). This resulted in ~60% of the dataset in study 1 labeled as “Fatigue” (~70% of the dataset in study 2).

### Task and gaze features

Study 1 used the following task-based features: object detection task accuracy, precision, recall, F1 score and the mean time to detect the target object. The gaze-based features were: gaze entropy (calculated as the Shannon entropy of the gaze heatmap), mean and standard deviation of the gaze error, and standard deviation of the gaze X and Y predictions. Similarly, for study 2, the task-based features were: typo detection accuracy, precision, recall and F1 score; while the gaze features were: saccade length, fixation duration, fixation frequency, X and Y view speed (total distance normalized by time) and reading speed (avg words read per minute). The divergence of these task and gaze features from normality was confirmed via the Shapiro–Wilk test. We used the two-tailed Wilcoxon signed-rank test to determine the statistical significance of the difference in these features between the "No Fatigue” and "Fatigue” blocks (Table 1).

### Model training

We use the scikit-learn SVC library (see sklearn.svm.SVC) for implementing this binary classification model. The precise mathematical formulation for the SVC we used can be found here: https://scikit-learn.org/stable/modules/svm.html#svc. Since we used a leave-one-user-out evaluation setting, a separate SVM model was trained per participant using the data instances from the remaining participants. We performed a randomized hyperparameter search (see sklearn.model_selection.RandomizedSearchCV) with *n* = 500 iterations for each user using the following as the parameter search space: kernel = [“linear”, “poly”, “rbf”, “sigmoid”], kernel coefficient *γ* = [10^−9^, 10^2^] ∪ [“scale”], regularization parameter C = [10^−3^, 10^2^] with the rest of the classifier parameters set to the implementation library (scikit-learn version 0.22) defaults. The best model hyperparameters were determined based on the average score after a three-fold cross-validation run on the training set.

### Reporting summary

Further information on research design is available in the Nature Research Reporting Summary linked to this article.

## Supplementary information

Reporting Summary

## Data Availability

To protect study participants’ privacy and consent, captured full face image data will not be publicly available. The de-identified gaze/task performance features, and corresponding fatigue labels for the studies are available upon reasonable request from the corresponding author V.N.

## Code availability

We made use of the open source machine learning frameworks TensorFlow (https://github.com/tensorflow) and scikit-learn (https://scikit-learn.org) to conduct our experiments. Due to the large number of dependencies on internal tooling, infrastructure and hardware, we are unable to publicly release this code. However, all the experiments and implementation details are available in the Methods section, to allow for independent replication.
