# Supplementary material for: Digital biomarker of mental fatigue
Source: NPJ Digit Med. 2021 Mar 11;4:47. doi: 10.1038/s41746-021-00415-6 (PMC7952693; doi:10.1038/s41746-021-00415-6)
Supplement: Supplementary file 1 — Reporting Summary [file 41746_2021_415_MOESM1_ESM.pdf]

nature research

Corresponding author(s): Vidhya Navalpakkam

Last updated by author(s): Aug 21, 2020

Reporting Summary

Nature Research wishes to improve the reproducibility of the work that we publish. This form provides structure for consistency and transparency in reporting. For further information on Nature Research policies, see Authors & Referees and the Editorial Policy Checklist.

Statistics

For all statistical analyses, confirm that the following items are present in the figure legend, table legend, main text, or Methods section.

n/a	Confirmed	
☐	☒	The exact sample size (*n*) for each experimental group/condition, given as a discrete number and unit of measurement
☐	☒	A statement on whether measurements were taken from distinct samples or whether the same sample was measured repeatedly
☐	☒	The statistical test(s) used AND whether they are one- or two-sided<br>*Only common tests should be described solely by name; describe more complex techniques in the Methods section.*
☐	☒	A description of all covariates tested
☐	☒	A description of any assumptions or corrections, such as tests of normality and adjustment for multiple comparisons
☐	☒	A full description of the statistical parameters including central tendency (e.g. means) or other basic estimates (e.g. regression coefficient) AND variation (e.g. standard deviation) or associated estimates of uncertainty (e.g. confidence intervals)
☐	☒	For null hypothesis testing, the test statistic (e.g. *F*, *t*, *r*) with confidence intervals, effect sizes, degrees of freedom and *P* value noted<br>*Give P values as exact values whenever suitable.*
☒	☐	For Bayesian analysis, information on the choice of priors and Markov chain Monte Carlo settings
☒	☐	For hierarchical and complex designs, identification of the appropriate level for tests and full reporting of outcomes
☒	☐	Estimates of effect sizes (e.g. Cohen's *d*, Pearson's *r*), indicating how they were calculated

Our web collection on statistics for biologists contains articles on many of the points above.

Software and code

Policy information about availability of computer code

Data collection	Android app for data collection (works on Android versions 8.1-9).
Data analysis	Gaze estimation and other analyses was done using TF2 with Keras and Scikit Learn (Python 3).<br>https://github.com/tensorflow/tensorflow<br>https://scikit-learn.org<br>Further data analysis used Python 3 and colab notebooks.

For manuscripts utilizing custom algorithms or software that are central to the research but not yet described in published literature, software must be made available to editors/reviewers. We strongly encourage code deposition in a community repository (e.g. GitHub). See the Nature Research guidelines for submitting code & software for further information.

Data

Policy information about availability of data

All manuscripts must include a data availability statement. This statement should provide the following information, where applicable:

- Accession codes, unique identifiers, or web links for publicly available datasets
- A list of figures that have associated raw data
- A description of any restrictions on data availability

To protect study participants' privacy and consent, captured full face image data will not be publicly available. The de-identified gaze/task performance measures, and corresponding fatigue labels for the studies are available upon reasonable request from the corresponding author V.N.

Field-specific reporting

Please select the one below that is the best fit for your research. If you are not sure, read the appropriate sections before making your selection.

☐ Life sciences ☒ Behavioural & social sciences ☐ Ecological, evolutionary & environmental sciences

For a reference copy of the document with all sections, see nature.com/documents/nr-reporting-summary-flat.pdf

Behavioural & social sciences study design

All studies must disclose on these points even when the disclosure is negative.

Study description	The goal of this study is to examine whether smartphone-based gaze can serve as an objective marker of mental fatigue. The studies in this manuscript are quantitative, and involved eye tracking as participants viewed stimuli on their phones. Studies were designed to be < 1 hour long and were conducted in lab settings.
Research sample	Similar to previous eye tracking studies in the literature, the sample was representative and spanned ages 18-55, including males and females with normal or corrected vision (no glasses). As described in the Methods section, participants were recruited from the local population in San Francisco Bay area.
Sampling strategy	Previous eye tracking studies have used a sample size of 2-30 participants. Examples below:<br>[1] Najemnik, Jiri, and Wilson S. Geisler. "Optimal eye movement strategies in visual search." Nature 434.7031 (2005): 387-391.<br>[2] Shimojo, Shinsuke, et al. "Gaze bias both reflects and influences preference." Nature neuroscience 6.12 (2003): 1317-1322.<br><br>Consistent with previous eye tracking studies, we used 24 and 27 participants for study 1 and study 2 respectively. As shown in the results section, we were able to achieve statistically significant results and test our hypotheses using this sample size.
Data collection	Data for all studies were collected using a custom-built Android app which ran on a Pixel 2 XL smartphone. For each study, data was collected in indoor settings with 5-6 participants seated in a conference room and a researcher providing instructions at the beginning. Researcher was blind to the experimental conditions and was not informed about the study hypothesis.
Timing	Both studies were conducted between July 23-26, 2019.
Data exclusions	As is common practice with eye tracking studies, we removed participants with high calibration error of over 2 degree viewing angle, as otherwise, the data is too noisy to make robust inferences on where the participant is looking. This translates to removing participants whose gaze error > 1cm (at a viewing distance of ~30cm from the screen). Further, participants who did not follow instructions or perform the task correctly, or did not respond for the entire duration of the fatigue-inducing tasks in the study were removed. After data cleaning, we have 17 and 15 participants for study 1 and study 2 respectively.
Non-participation	No participants dropped out or declined participation.
Randomization	Participants were not allocated into experimental groups. Studies had a within-subject design, and each participant performed tasks under all experimental conditions, in randomized order.

Reporting for specific materials, systems and methods

We require information from authors about some types of materials, experimental systems and methods used in many studies. Here, indicate whether each material, system or method listed is relevant to your study. If you are not sure if a list item applies to your research, read the appropriate section before selecting a response.

Materials & experimental systems

n/a	Involved in the study	
☒	☐	Antibodies
☒	☐	Eukaryotic cell lines
☒	☐	Palaeontology
☒	☐	Animals and other organisms
☐	☒	Human research participants
☒	☐	Clinical data

Methods

n/a	Involved in the study	
☒	☐	ChIP-seq
☒	☐	Flow cytometry
☒	☐	MRI-based neuroimaging

Human research participants

Policy information about studies involving human research participants

Population characteristics	See above.
Recruitment	Participants were randomly selected from a pool of user study volunteers who signed up through the Google User Experience Research portal. To minimize self-selection bias, we selected a sample that was representative and diverse and spanned a wide age range (18-55), gender (both males and females), diverse professions.
Ethics oversight	This study was reviewed by the Advarra Institutional Review Board (IRB) (Columbia, MD) and determined to be exempt from IRB oversight. This work has been performed in accordance with relevant guidelines and regulations, and approved by Google AI principles team. The data was collected with participants' consent in accordance with the Google Privacy and Legal Policy. We added this to the manuscript.

Note that full information on the approval of the study protocol must also be provided in the manuscript.
